# Role of *Lactobacillus reuteri* in Human Health and Diseases

**DOI:** 10.3389/fmicb.2018.00757

**Published:** 2018-04-19

**Authors:** Qinghui Mu, Vincent J. Tavella, Xin M. Luo

**Affiliations:** Department of Biomedical Sciences and Pathobiology, Virginia-Maryland College of Veterinary Medicine, Virginia Tech, Blacksburg, VA, United States

**Keywords:** *Lactobacillus reuteri*, probiotic, microbiota, immune system, inflammatory diseases

## Abstract

*Lactobacillus reuteri* (*L. reuteri*) is a well-studied probiotic bacterium that can colonize a large number of mammals. In humans, *L. reuteri* is found in different body sites, including the gastrointestinal tract, urinary tract, skin, and breast milk. The abundance of *L. reuteri* varies among different individuals. Several beneficial effects of *L. reuteri* have been noted. First, *L. reuteri* can produce antimicrobial molecules, such as organic acids, ethanol, and reuterin. Due to its antimicrobial activity, *L. reuteri* is able to inhibit the colonization of pathogenic microbes and remodel the commensal microbiota composition in the host. Second, *L. reuteri* can benefit the host immune system. For instance, some *L. reuteri* strains can reduce the production of pro-inflammatory cytokines while promoting regulatory T cell development and function. Third, bearing the ability to strengthen the intestinal barrier, the colonization of *L. reuteri* may decrease the microbial translocation from the gut lumen to the tissues. Microbial translocation across the intestinal epithelium has been hypothesized as an initiator of inflammation. Therefore, inflammatory diseases, including those located in the gut as well as in remote tissues, may be ameliorated by increasing the colonization of *L. reuteri*. Notably, the decrease in the abundance of *L. reuteri* in humans in the past decades is correlated with an increase in the incidences of inflammatory diseases over the same period of time. Direct supplementation or prebiotic modulation of *L. reuteri* may be an attractive preventive and/or therapeutic avenue against inflammatory diseases.

## Introduction

Probiotics are defined as “live microorganisms which, when administered in adequate amounts, confer a health benefit on the host” by the World Health Organization. While the idea to use probiotics for health benefits is not new, the interest has significantly increased in recent years (Islam, 2016). This may be due, in part, to the increase in antibiotic resistance particularly in the treatment of diseases in the gastrointestinal (GI) system, as well as an increasing desire by the public for natural health promotants. Those probiotic microorganisms that have been shown to have beneficial properties include *Lactobacillus spp., Bifidobacterium spp., Saccharomyces boulardii, Propionibacterium spp., Streptococcus spp., Bacillus spp., Enterococcus spp.*, and some specific strains of *Escherichia coli* (Kechagia et al., 2013).

There are certain criteria that a probiotic must have to be considered efficacious. These include the capacity to survive in the GI tract, a high resistance to gastric acids, the lack of any transferable antibiotic resistance genes, and the capacity to exert clear benefits in the host (Montalban-Arques et al., 2015). Probiotics promote a healthy body through diverse mechanisms. A widespread generalization describing common mechanisms among studied probiotic genera includes colonizing resistance, producing acid, and short chain fatty acid (SCFA), regulating intestinal transit, normalizing perturbed microbiota, increasing enterocyte turnover, and competitive exclusion of pathogens (Hill et al., 2014). Though not widely observed, there are a lot of effects among specific probiotic species, some being strain specific. For instance, some probiotic strains can improve host food digestion by metabolizing bile salt or complementing the functions of missing digestive enzymes (Amara and Shibl, 2015; Shi et al., 2016).

*Lactobacillus* spp. are one of the most widely used probiotics and can be found in a large variety of food products throughout the world (Giraffa et al., 2010). The genus *Lactobacillus* comprises a large heterogeneous group of Gram-positive, nonsporulating, facultative anaerobic bacteria which include *L. acidophilus, L. rhamnosus, L. bulgaricus, L. casei*, and *L. reuteri*. This genus plays a very important role in food fermentation and can also be found in the GI system of humans and animals in variable amounts depending on the species, age of the host, or location within the gut (Duar et al., 2017).

Animal studies and preclinical results have shown that *Lactobacilli* may help in the prevention and treatment of numerous GI tract disorders. Among these disorders are enteric infections, antibiotic-associated diarrhea, necrotizing enterocolitis in preterm neonates, inflammatory bowel disease, colorectal cancer, and irritable bowel syndrome (Lebeer et al., 2008). Although the GI tract is the site where *Lactobacilli* are believed to show the most benefits, probiotic applications of some *Lactobacillus* strains at other sites of the body have been reported. These include the prevention and treatment of urogenital diseases and bacterial vaginosis in women, atopic disease, food hypersensitivity, and the prevention of dental caries (Lebeer et al., 2008).

One species of *Lactobacillus, L. reuteri* has multiple beneficial effects on host health such as prevention and/or amelioration of diverse disorders. *L. reuteri* was first isolated in 1962. It has been characterized as heterofermentative species that grows in oxygen-limited atmospheres and colonizes the GI tract of humans and animals (Kandler et al., 1980). The fact that it normally colonizes the GI tract may be the reason it confers great probiotic properties. This organism can withstand a wide variety of pH environments, employs multiple mechanisms that allow it to successfully inhibit pathogenic microorganisms, and has been shown to secrete antimicrobial intermediaries (Jacobsen et al., 1999; Valeur et al., 2004).

*L. reuteri* has been shown to be one of the truly indigenous bacteria of the human GI tract (Sinkiewicz, 2010). It naturally colonizes a wide range of vertebrates, including pigs, rodents, and chickens. In fact, it has gone through long-term evolution to diversify into host-adapted lineages (Oh et al., 2010; Walter et al., 2011). This organism is most typically found in the proximal digestive tract of the host (Frese et al., 2013). Several studies have assessed the safety of this organism in adults, children, infants, and even in an HIV-infected population (Wolf et al., 1998; Valeur et al., 2004; Weizman and Alsheikh, 2006; Mangalat et al., 2012; Jones et al., 2012a,c; Hoy-Schulz et al., 2016). The results showed that a dose as high as 2.9 × 10^9^ colony-forming units (cfu)/day was still well tolerated, safe, and efficacious in humans. There have also been numerous articles enumerating the benefits of *L. reuteri* as a probiotic. These benefits include promoting health, reducing infections, improving feed tolerance, enhancing the absorption of nutrients, minerals, and vitamins, modulating host immune responses, promoting gut mucosal integrity, and reducing bacterial translocation (Tubelius et al., 2005; McFall-Ngai, 2007; Indrio et al., 2008; Spinler et al., 2008; Hou et al., 2015). In the current review, we will focus on the particular probiotic, *L. reuteri*, and discuss its beneficial functions in promoting health and preventing infections and diverse diseases.

## Probiotic Properties of *L. reuteri*

**FIGURE 1:**
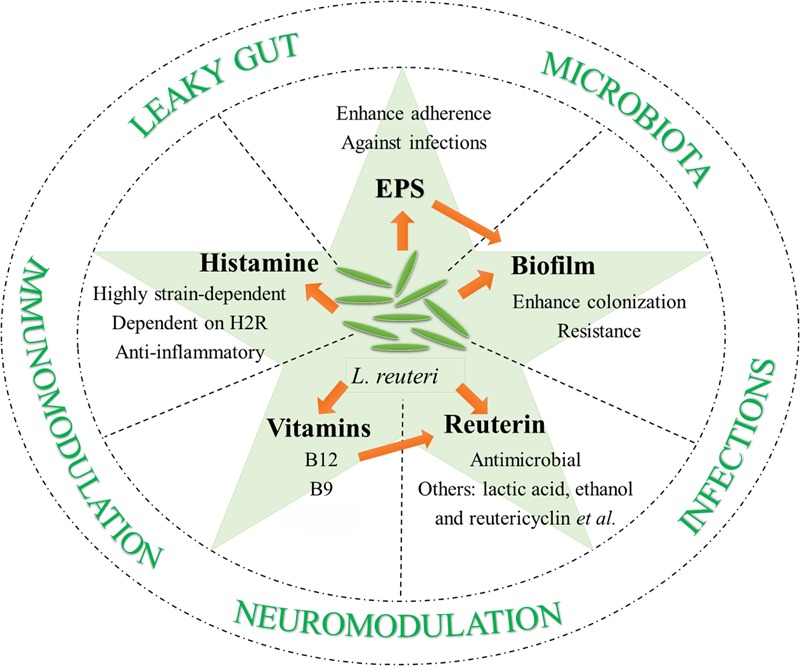
Probiotic properties of *L. reuteri*.

There are some prerequisites for becoming potential probiotics: to survive in low pH and enzyme-enriched environments, to adhere to epithelium for host-probiotic interaction, competition with pathogenic microorganisms, and most importantly, safety. *L. reuteri* meets all of these requirements. Here, additional probiotic properties of *L. reuteri* are discussed that contribute to its diverse beneficial effects on host health and disease prevention and/or amelioration (**Figure 1**).

### Gut Colonization of *L. reuteri*

Built for digestion and absorption, some sites of the GI system have developed to be harsh for microorganism colonization. Examples of this can be seen in the low pH conditions caused by gastric acids and bile salts in upper small intestine. Thus, the very first step of colonizing the GI tract is to survive in such environments. Multiple *L. reuteri* stains are resistant to low pH and bile salts (Seo et al., 2010; Krumbeck et al., 2016). This resistance is believed to be at least partially dependent on its ability to form biofilms (Salas-Jara et al., 2016).

*L. reuteri* is capable of attaching to mucin and intestinal epithelia, and some strains can adhere to gut epithelial cells in a range of vertebrate hosts (Li et al., 2008; Hou et al., 2014, 2015). A possible mechanism for adherence is the binding of bacterial surface molecules to the mucus layer. Mucus-binding proteins (MUBs) and MUB-like proteins, encoded by *Lactobacillales*-specific clusters of orthologous protein coding genes, serve as adherence mediators, or so-called adhesins (Roos and Jonsson, 2002; Kleerebezem et al., 2010; Gunning et al., 2016). The considerable diversity of MUBs among *L. reuteri* strains and the variation in the abundance of cell-surface MUBs significantly correlates with their mucus binding ability (Mackenzie et al., 2010). The strain-specific role of MUBs in recognizing mucus elements and/or their capability of promoting aggregation can explain the contribution of MUBs on the adherence of *L. reuteri*. Factors that mediate the attachment to the surfaces include multiple large surface proteins (Walter et al., 2005; Wang et al., 2008; Frese et al., 2011), MUB A (Jensen et al., 2014), glucosyltransferase A (GtfA) and inulosucrase (Inu) (Walter et al., 2008), and D-alanyl ester (Walter et al., 2007).

As *L. reuteri* that has colonized to the host GI tract can form biofilms, efforts have been made to study the regulation of *L. reuteri* biofilm secretion and its association with the adherence of bacteria to host GI epithelium. By doing *in vitro* biofilm assay, Water, J. et al. uncovered the contribution of GtfA and Inu in the biofilm formation of *L. reuteri* TMW1.106 (Walter et al., 2008). The *in vivo* biofilm formation of *L. reuteri* strains seems to be dependent on the host origin of the strains. In one study, nine *L. reuteri* strains isolated from different hosts (human, mouse, rat, chicken, and pig) were given to germ-free mice and the biofilms were evaluated after 2 days. Interestingly, only rodent strains were able to form biofilms and adhere to the forestomach epithelium, although the luminal populations were comparable among strains of different origins (Frese et al., 2013). Another study by the same authors showed that a specialized transport pathway (the SecA2-SecY2 system) was unique in the rodent and porcine strains (Frese et al., 2011). By using a rodent strain *L. reuteri* 100-23, they compared extracellular and cell wall-associated proteins between the wild-type strain and the *secA2* mutant. Only one surface protein, *L. reuteri* 70902, was absent in the *secA2* mutant. *In vivo* colonization studies showed that the absence of *L. reuteri* 70902 leads to almost completely eliminated biofilm formation. This strongly suggests that *L. reuteri* 70902 and the SecA2-SecY2 system are key factors regulating biofilm production from *L. reuteri* 100-23 in germ-free mice (Frese et al., 2013). Another group investigated the role of two-component systems *bfrKRT* and *cemAKR* in *in vitro* biofilm formation of *L. reuteri* 100-23 (Su and Ganzle, 2014). They found the deletion of certain genes in the operons enhanced the adherence and biofilm formation. However, the contribution of the *bfrKRT* and *cemAKR* to *in vivo* biofilm formation remains to be elucidated. The role of exopolysaccharide (EPS) in assisting colonization was also examined with *L. reuteri* 100-23. The production of EPS was eliminated due to a mutation of the fructosyl transferase (*ftf*) gene (Sims et al., 2011). After administration to *Lactobacillus*-free mice, compared to the wild-type strain, the colonization of the *ftf* mutant in the forestomach and cecum was largely impaired. This indicates EPS production can enhance the colonizing ability of strain 100-23 in the gut. Interestingly, *L. reuteri* RC-14 has been demonstrated to be able to penetrate mature *E. coli* biofilm and become part of it (McMillan et al., 2011). Recently, *L. reuteri* was delivered as a biofilm on microsphere and such delivery was found to promote the adherence of *L. reuteri* to intestinal epithelium and enhance its probiotic property (Olson et al., 2016; Navarro et al., 2017).

### Production of Metabolites With Health-Promoting Effect

The antimicrobial and immunomodulatory effects of *L. reuteri* strains are linked to their metabolite production profile. Here, we discuss a few well-studied metabolites with regard to the probiotic potential of *L. reuteri*.

#### Reuterin

Most *L. reuteri* strains of human and poultry lineage are able to produce and excrete reuterin, a well-known antimicrobial compound (Talarico et al., 1988; Talarico and Dobrogosz, 1989; Cadieux et al., 2008; Jones and Versalovic, 2009; Mishra et al., 2012; Greifova et al., 2017). Reuterin is a mixture of different forms of 3-hydroxypropionaldehyde (3-HPA) (Talarico and Dobrogosz, 1989). It is known that *L. reuteri* can metabolize glycerol to generate 3-HPA in a coenzyme B12-dependent, glycerol dehydratase-mediated reaction (Talarico and Dobrogosz, 1990; Chen and Chen, 2013). The production of 3-HPA has also been demonstrated in a few other bacterial species (Zhu et al., 2002; Raynaud et al., 2003; Yang et al., 2007). However, *L. reuteri* is unique in its ability to produce and secrete 3-HPA in a manner more than its bioenergetics requirement (Stevens et al., 2011). Moreover, the antimicrobial activity of reuterin seems to rely on the spontaneous conversion of 3-HPA to acrolein, a cytotoxic electrophile (Stevens and Maier, 2008; Engels et al., 2016). Reuterin can inhibit a wide range of microorganisms, mainly Gram-negative bacteria (Cleusix et al., 2007). Not surprisingly, most *Lactobacillus* species are resistant to reuterin, among which *L. reuteri* strains exert the most resistance (Jones and Versalovic, 2009; Mishra et al., 2012). In addition to its antimicrobial property, reuterin is able to conjugate heterocyclic amines, which also seems to be dependent on the formation of acrolein (Engels et al., 2016). This suggests acrolein is an essential compound in the activity of reuterin.

Apart from reuterin, several other antimicrobial substances, including lactic acid, acetic acid, ethanol, and reutericyclin, have been determined as products of some *L. reuteri* strains (Ganzle and Vogel, 2003; Burge et al., 2015; Gopi et al., 2015; Yang Y. et al., 2015; Greifova et al., 2017). With the synthesis of these substances, *L. reuteri* has been shown to be effective against a variety of GI bacterial infections. These infections include *Helicobacter pylori, E. coli, Clostridium difficile*, and *Salmonella* (Reid and Burton, 2002; Cherian et al., 2015; Abhisingha et al., 2017; Genis et al., 2017). One of the more notable illustrations of the efficacy of *L. reuteri* as a probiotic against infections is the use of *L. reuteri* to treat *H. pylori*. *H. pylori* infection is a major cause of chronic gastritis and peptic ulcers, as well as a risk factor for gastric malignancies (Franceschi et al., 2007; Lesbros-Pantoflickova et al., 2007; Park et al., 2007). The use of *L. reuteri* against *H. pylori* has been explored in many studies (**Table 1**). It has been suggested that *L. reuteri* works by competing with *H. pylori* and inhibiting its binding to glycolipid receptors (Mukai et al., 2002). The competition reduces the bacterial load of *H. pylori* and decreases the related symptoms (Lionetti et al., 2006; Francavilla et al., 2008). Some studies have shown that *L. reuteri* has the potential to completely eradicate *H. pylori* from the intestine (Ojetti et al., 2012). Importantly, *L. reuteri* is advantageous in the treatment of *H.* pylori as the supplementation eradicates the pathogen without causing the common side effects associated with antibiotic therapies (Francavilla et al., 2014).

**Table 1:** Clinical efficacies of *L. reuteri* against *H. pylori.*

Strain	Treatment	Subjects	Result	Citation
DSM 17648	14 days	Adults	Decrease in pathogen load in the stomach	Holz et al., 2015
DSM 17938	20 days	Patients	93% successful eradication of the pathogen with inhibitor-tetracycline-metronidazole – *L. reuteri* therapy	Dore et al., 2016
ATCC 55730	10 days	Infected children	Improvement of GI symptoms	Lionetti et al., 2006
–	7 days	Patients	No improvement of the standard triple therapy	Scaccianoce et al., 2008
ATCC 55730	4 weeks	Patients	Significant decrease of pathogen load and improvement of dyspeptic symptoms	Francavilla et al., 2008
SD2112	4 weeks	Patients	Decrease of pathogen density and suppression of urease activity	Imase et al., 2007
DSMZ 17648	14 days	Patients	Decrease in pathogen load	Mehling and Busjahn, 2013
DSM 17938, ATCC PTA 6475	During therapy	Patients	Reduction of antibiotic-associated side effects in eradication therapy	Francavilla et al., 2014
DSM 17938	8 weeks	Patients	Decrease of urease activity in pantoprazole therapy	Dore et al., 2014

A considerable amount of research has been done to determine the beneficial effects of *L. reuteri* against viruses and/or fungi. There is evidence showing the benefit of *L. reuteri* against pneumoviruses, circoviruses, rotaviruses, coxsackieviruses, and papillomaviruses (Shornikova et al., 1997a,b; Preidis et al., 2012; Ang et al., 2016; Brenner et al., 2016; Piyathilake et al., 2016; Karaffova et al., 2017). It has been suggested that *L. reuteri* ameliorates viral infection by regulating the microbiota and secreting metabolites that have antiviral components (Ang et al., 2016). Furthermore, some studies suggest that *L. reuteri* may have antifungal properties as well, where *L. reuteri* antagonizes, stops the growth of, and eventually kills various species of Candida (Jorgensen et al., 2017).

#### Histamine

A few strains of *L. reuteri* are able to convert the amino acid L-histidine, a dietary component, to the biogenic amine histamine (Diaz et al., 2016; Greifova et al., 2017). A human commensal bacterium, *L. reuteri* 6475 was used as the model strain for studying histamine in *L. reuteri*. J. Versalovic’s group reported that *L. reuteri* 6475-derived histamine suppressed tumor necrosis factor (TNF) production from stimulated human monocytes (Thomas et al., 2012). This suppression was dependent on the activation of histamine H_2_ receptor, increased intracellular cAMP and protein kinase A, and the inhibition of MEK/ERK signaling. The production of histamine and subsequent *in vitro* TNF-suppressing function are regulated by a complete chromosomal histidine decarboxylase (*hdc*) gene cluster, which contains *hdcA, hdcB*, and *hdcP* (Rossi et al., 2011; Thomas et al., 2012). The same group of researchers also found that oral administration of *hdc*^+^ *L. reuteri* could effectively suppress intestinal inflammation in a trinitrobenzene sulfonic acid (TNBS)-induced mouse colitis model (Gao et al., 2015). Moreover, intraperitoneal injection of *L. reuteri* 6475 culture supernatant to TNBS-treated mice resulted in similar colitis attenuation. These results strongly indicate the involvement of *L. reuteri* metabolites, including histamine, in intestinal immunomodulation (Thomas et al., 2016). Further investigations showed that a gene called *rsiR* was necessary for the expression of *hdc* gene cluster in *L. reuteri* 6475 (Hemarajata et al., 2013). Inactivation of *rsiR* gene led to reduced TNF inhibition *in vitro* and diminished anti-inflammatory function *in vivo*. Additionally, both the *in vitro* TNF suppression and the *in vivo* anti-colitis effects appear to be regulated by a gene named *folC2* (Thomas et al., 2016). Inactivation of *folC2* gene resulted in suppression of the *hdc* gene cluster and diminished histamine production. Notably, histamine production by *L. reuteri* is highly strain-dependent, and most studies have been focused on strains of human origin (Mishra et al., 2012).

#### Vitamins

There are 13 essential vitamins for humans due to the inability of the human body to synthesize them (Linares et al., 2017). Like many other *Lactobacillus* spp., several *L. reuteri* strains are able to produce different types of vitamins, including vitamin B12 (cobalamin) and B9 (folate). As mentioned earlier, B12 is vital in reuterin production because the reduction of glycerol to 3-HPA requires a B12-dependent coenzyme. Up to now, at least 4 *L. reuteri* strains with various origins have been found to produce B12 (Taranto et al., 2003; Santos et al., 2008b; Sriramulu et al., 2008; Gu et al., 2015). Among these strains, *L. reuteri* CRL1098 and *L. reuteri* JCM1112 are the most studied (Morita et al., 2008; Santos et al., 2008a, 2011). In one study, the administration of *L. reuteri* CRL1098 together with a diet lacking vitamin B12 was shown to ameliorate pathologies in B12-deficient pregnant female mice and their offspring (Molina et al., 2009). This clearly points to the potential application of *L. reuteri* in treating B12 deficiency. In addition to B12, folate can also be synthesized by some specific *L. reuteri* strains, including *L. reuteri* 6475 and *L. reuteri* JCM1112 (Santos et al., 2008b; Thomas et al., 2016).

#### Exopolysaccharide (EPS)

The EPS produced by *L. reuteri* is important for biofilm formation and adherence of *L. reuteri* to epithelial surfaces (Salas-Jara et al., 2016). In addition, EPS synthesized by *L. reuteri* is able to inhibit *E. coli* adhesion to porcine epithelial cells *in vitro* (Ksonzekova et al., 2016). More importantly, EPS-mediated blocking of adhesion also suppresses gene expression of pro-inflammatory cytokines that are induced by *E. coli* infection, including IL-1β and IL-6. Further *in vivo* experiments in piglets showed similar results in that EPS originated from *L. reuteri* prevented piglet diarrhea in bacterial infection by reducing the adhesion of *E. coli* (Chen et al., 2014). In addition, EPS of *L. reuteri* origin has been reported to suppress the binding of enterotoxigenic *E. coli* to porcine erythrocytes (Wang et al., 2010). EPS produced by rodent *L. reuteri* 100-23 was also demonstrated to induce Foxp3^+^ regulatory T (Treg) cells in the spleen (Sims et al., 2011). In contrast, an *L. reuteri* 100-23 strain with the *ftf* mutation that eliminates EPS production from *L. reuteri* did not induce splenic Treg cells. This suggests that EPS is required for the *L. reuteri*-mediated induction of Treg cells and indicates the potential of using wild-type *L. reuteri* 100-23 to treat Treg deficiency.

### *L. reuteri*-Mediated Modulation of Host Microbiota

Emerging evidence suggests that the host microbiota and immune system interact to maintain tissue homeostasis in healthy individuals (Kamada et al., 2013; Bene et al., 2017). Many diseases have been associated with perturbation of the microbiota (Mu et al., 2015), whereas restoration of the microbiota has been demonstrated to prevent or ameliorate several diseases (Scott et al., 2015). *L. reuteri* is able to influence the diversity, composition and metabolic function of the gut, oral, and vaginal microbiotas. These effects are largely strain-specific (Yang Y. et al., 2015; Garcia Rodenas et al., 2016; Galley et al., 2017; Su et al., 2017).

#### Gut Microbiota

Studies have shown the modulatory effects of *L. reuteri* on the microbiotas of rodents, piglets, and humans. One study assessed oral administration of a human-origin *L. reuteri* strain (DSM17938) to scurfy mice, which have gut microbial dysbiosis due to the *foxp3* gene mutation. The results indicated that this strain of *L. reuteri* was able to prolong the lifespan of the mice and reduce multi-organ inflammation while remodeling the gut microbiota (He et al., 2017). Changes of gut microbiota included increases in the phylum *Firmicutes* and the genera *Lactobacillus* and *Oscilospira*. Notably, the disease-ameliorating effect of *L. reuteri* was attributed to the remodeled gut microbiota, though the community composition was still distinct from wild-type littermates. Further investigation showed that inosine production was enhanced by the gut microbiota upon *L. reuteri* administration. Through adenosine A_2A_ receptor engagement, inosine can reduce Th1/Th2 cells and their associated cytokines. These results suggested that the *L. reuteri* – gut microbiota – inosine – adenosine A_2A_ receptor axis serves as a potential therapeutic method for Treg-deficient disorders. Moreover, oral *L. reuteri* 6475 treatment led to a higher diversity of microbiota in both jejunum and ileum in an ovariectomy-induced bone loss mouse model (Britton et al., 2014). Specifically, there were more abundant *Clostridiales* but less *Bacteriodales*. However, whether or not the changed gut microbiota was directly associated with the prevention of bone loss requires further investigation. Furthermore, *L. reuteri* C10-2-1 has been shown to modulate the diversity of gut microbiota in the ileum of rats (Wang P. et al., 2016).

Compared to vaginally delivered infants, Cesarean (C)-section delivered infants display a higher abundance of *Enterobacter* but less *Bifidobacterium* in their gut microbiota (Garcia Rodenas et al., 2016; Nagpal et al., 2016). In one study, treating C-section babies with *L. reuteri* DSM 17938 from 2 weeks to 4 months of age modulated the development of gut microbiota toward the community pattern found in vaginally delivered infants (Garcia Rodenas et al., 2016). The gut microbiota structure of vaginally born infants remained unaltered upon *L. reuteri* supplementation. In another study, treating infants with the same *L. reuteri* strain resulted in decreased anaerobic Gram-negative and increased Gram-positive bacterial counts in gut microbiota, whereas the abundances of *Enterobacteriaceae* and *Enterococci* were largely lowered by *L. reuteri* treatment (Savino et al., 2015b). The differences in infant age, duration of treatment, route of administration, and dosage may explain the differences in results from the two studies.

For human adults, *L. reuteri* NCIMB 30242 administered as delayed release capsules for 4 weeks was able to increase the ratio of *Firmicutes* to *Bacteroidetes* in healthy individuals (Martoni et al., 2015). This strain of *L. reuteri* is known to be able to activate bile salt hydrolase and its effect in increasing circulating bile acid has been reported (Jones et al., 2012b). The upregulation of circulating bile acid has been proposed as a reason for the modulated gut microbiota (Jones et al., 2012b). In type 2 diabetes patients, although 3 months of *L. reuteri* DSM 17938 supplementation did not significantly change the gut microbial structure, the disease outcome of *L. reuteri* treatment was highly correlated with the baseline gut microbiota structure of individuals (Mobini et al., 2017). Furthermore, the administration of *L. reuteri* DSM 17938 in cystic fibrosis (CF) patients rescued gut microbiota dysbiosis by reducing *Proteobacteria* while also enhancing the relative abundance of *Firmicutes* (del Campo et al., 2014). However, whether or not the modulated gut microbiota contributed to improved GI health in probiotic-treated CF patients needs to be explored further.

*L. reuteri* influences the gut microbial community in piglets in a strain-specific manner. For instance, oral *L. reuteri* ZLR003 administration was able to change both the diversity and the composition of the gut microbiota (Zhang et al., 2016). However, treatment with the I5007 strain did not affect colonic microbial structure in piglets (Liu H. et al., 2017). In another study, fodder fermented with *L. reuteri* changed the abundances of 6 different bacterial taxa, particularly the family *Enterobacteriacae*, in weanling pigs (Yang Y. et al., 2015). However, the major alterations including increased *Mitsuokella* and decreased a family under phylum *Bacteroidetes* could only be seen with *L. reuteri* TMW1.656 rather than *L. reuteri* LTH5794. TMW1.656 is a reutericyclin-producing strain while LTH5794 is not, suggesting the possible contribution of reutericyclin in modulating gut microbiota in piglets (Yang Y. et al., 2015).

#### Oral Microbiota

The phyla *Firmicutes, Bacteroidetes, Fusobacteria, Proteobacteria, and Actinobacteria* are most abundant in the human oral microbiome (Romani Vestman et al., 2015). In a randomized controlled trial, 12 weeks of daily consumption of two *L. reuteri* strains – DSM 17938 and PTA 5289 led to a shift in oral microbiota composition, though the bacterial species richness was not altered (Romani Vestman et al., 2015). The alterations disappeared 4 weeks after the treatments were terminated, suggesting the fast turnover of the oral microbiome. In another human study, oral *L. reuteri* treatment reduced the amount of periodontal pathogens in the subgingival microbiota, though no clinical impact was seen (Iniesta et al., 2012).

#### Vaginal Microbiota

*Lactobacilli* dominate the vaginal bacterial community in healthy women (Macklaim et al., 2015). One study showed that only 14 days of oral *L. reuteri* RC-14 administration could restore the normal vaginal flora in postmenopausal women (Petricevic et al., 2008). Interestingly, the relative abundance of *Lactobacilli* is largely decreased in bacterial vaginosis patients (Macklaim et al., 2015). A total of 4 weeks of oral capsule consumption of two *Lactobacilli* strains including *L. reuteri* RC-14 increased the relative abundance of *Lactobacilli*. A similar increase of *Lactobacilli* was seen when *L. reuteri* RC-14 was administered vaginally together with a *L. rhamnosus* strain (Bisanz et al., 2014). However, in pregnant women, 8 weeks of oral *L. reuteri* RC-14 treatment did not efficiently restore the normal vaginal microbiota (Gille et al., 2016). This suggests that *L. reuteri* RC-14 may not be able to act alone.

### Role of *L. reuteri* in Immunomodulation

*Lactobacillus reuteri* is able to increase free secretory IgA (sIgA) levels in rats (Wang P. et al., 2016). However, the upregulation of sIgA was eliminated in vitamin A-deficient rats, suggesting that *L. reuteri* functions in a vitamin A-dependent manner. In pregnant women, the intake of *L. reuteri* did not alter the levels of total IgA or sIgA in breast milk (Bottcher et al., 2008). When it comes to the effect of *L. reuteri* in inducing salivary IgA, the results are controversial. Increased salivary IgA levels were reported in humans’ chewing gum containing *L. reuteri* (Ericson et al., 2013). However, other studies showed that *L. reuteri* did not affect IgA concentration in saliva (Garofoli et al., 2014; Jorgensen et al., 2016; Braathen et al., 2017). The difference in the strains of *L. reuteri* used in the studies may explain the difference in results. Notably, an important commonality is that salivary *L. reuteri*-positive individuals have higher salivary IgA levels. Whether *L. reuteri* affects IgA levels by directly regulating B cells requires further investigations.

Many studies have shown that *L. reuteri* can induce anti-inflammatory Treg cells, which likely contributes to the beneficial effects of *L. reuteri* in a wide range of diseased and non-diseased conditions (**Table 2**). The Treg-inducing property of *L. reuteri* is largely strain-dependent. However, the anti-inflammatory effect of *L. reuteri* does not always rely on the induction of Treg cells. A good example is *L. reuteri*-mediated suppression of Th1/Th2 responses in Treg-deficient mice (He et al., 2017). Certain *L. reuteri* strains are able to reduce the production of many pro-inflammatory cytokines. For example, *L. reuteri* GMNL-263 can reduce serum MCP-1, TNF, and IL-6 levels in mice fed with high fat diet (Hsieh et al., 2016). Similar effects were observed in mice treated with heat-killed GMNL-263. However, in some cases, the immunomodulatory effects of *L. reuteri* appear to rely on its metabolites, as the culture supernatant of *L. reuteri* BM36301 could reduce TNF production from human myeloid THP-1 cells (Lee et al., 2016). Interestingly, tryptophan catabolites of *L. reuteri* have been recognized as ligands for aryl hydrocarbon receptor (AhR). Through activating AhR, *L. reuteri* can promote local IL-22 production from innate lymphoid cells (ILCs) (Zelante et al., 2013). In addition, the derivatives of tryptophan generated by *L. reuteri* can induce the development of regulatory CD4^+^CD8αα^+^ double-positive intraepithelial lymphocytes in an AhR-dependent manner (Cervantes-Barragan et al., 2017). Considering that AhR is ubiquitously expressed, *L. reuteri* and its metabolites may be able to influence many more types of immune cells beyond ILCs and T cells (Nguyen et al., 2013).

**Table 2:** *L. reuteri*-mediated induction of Treg cells under various diseased and non-diseased conditions.

Condition	Subject	Tissue	Strain	Citation
Western-diet-associated obesity	Mouse	MLN	ATCC PTA 6475	Poutahidis et al., 2013b
Wound healing	Mouse	Biopsy	ATCC PTA 6475	Poutahidis et al., 2013a
Systemic lupus erythematosus	Mouse	Kidney	ATCC PTA 6475	Mu et al., 2017c
Necrotizing enterocolitis	Mouse	Intestine, MLN	DSM 17938	Liu et al., 2013, 2014
Wild-type	Mouse	MLN, Spleen	100-23	Livingston et al., 2010; Sims et al., 2011
Wild-type	Mouse	Spleen	ATCC 23272	Karimi et al., 2009
Wild-type, IBD, atopic dermatitis	Mouse	MLN, Colon, Ear	–	Abediankenari and Ghasemi, 2009
IBD	Human	Peripheral blood	RC-14	Lorea Baroja et al., 2007

*IBD, inflammatory bowel disease; MLN, mesenteric lymph node.*

### Neuromodulatory Capability of *L. reuteri*

The intestinal microbiota plays a role in the functions of the enteric nervous system (ENS) (Yoo and Mazmanian, 2017). Subjects with microbiota depletion exhibit an abnormal ENS state (Anitha et al., 2012; Brun et al., 2013, 2015; Yoo and Mazmanian, 2017). Antibiotic treatment reduces the number of neurons in the ENS. This may be related to the decrease in Glial cell line-derived neurotrophic factor (GDNF), which can be restored by TLR2 stimulation (Brun et al., 2013). Moreover, germ-free animals show defective ENS morphology and excitability, which can be reversed by microbiota colonization (McVey Neufeld et al., 2013; Collins et al., 2014). *L. reuteri*, specifically, can prevent visceral pain response mainly by reducing the enteric nerve activity during the colorectal distension pressure in mice (Kamiya et al., 2006; Ma et al., 2009). Interestingly, live, heat-killed, gamma-irradiated *L. reuteri*, or even the conditioned media all had a similar effect (Kamiya et al., 2006). *L. reuteri* can also produce gamma-aminobutyric acid (GABA), the major inhibitory neurotransmitter in the central nervous system (Su et al., 2011; Barrett et al., 2012; Pallin et al., 2016). However, the *in vivo* bioactivity of the produced GABA has not been addressed (Yoo and Mazmanian, 2017). Furthermore, *L. reuteri* can increase the excitability and the number of action potentials in rat colonic sensory neurons (Kunze et al., 2009). These distinct effects of *L. reuteri* may be due to the difference in target neurons (Lai et al., 2017).

### Role of *L. reuteri* in Reversing the Leaky Gut

Physical, biochemical, and immunological barriers comprise the gut barrier function, which is required to block the entry of exterior antigens and toxins (Mu et al., 2017a). If any abnormalities occur in the intestinal barrier, the permeability may increase resulting in a leaky gut. Various probiotics are known for their abilities to enhance mucosal barrier function, of which *L. reuteri* is a well-known example (Mu et al., 2017a). In DSS-induced colitis, *L. reuteri* administration could reduce bacterial translocation from the GI tract to the mesenteric lymph nodes (MLN) (Dicksved et al., 2012). In addition, treatment of lupus-prone mice with a mixture of *Lactobacillus* species including *L. reuteri* led to a higher expression of tight junction (TJ) proteins in intestinal epithelial cells (Mu et al., 2017c). Subsequently, the translocation of pro-inflammatory molecules such as LPS was significantly suppressed, which correlated with attenuated disease. In addition to mouse studies, several strains of *L. reuteri* have been shown to possess the ability to modulate TJ protein expression and maintain intestinal barrier integrity in pigs (Yang F. et al., 2015; Wang Z. et al., 2016). Moreover, the ability of *L. reuteri* to decrease intestinal permeability has been seen in humans. In children with atopic dermatitis, where the impairment of intestinal barrier function has been positively correlated with disease pathogenesis (De Benedetto et al., 2011), treatment with *L. reuteri* DSM 12246 (and *L. rhamnosus* 19070-2) significantly reduced the frequency of GI symptoms while decreasing the lactulose to mannitol ratio (Rosenfeldt et al., 2004), which reflects the reversal of a leaky gut (Camilleri et al., 2010).

## *L. reuteri* Attenuate Human Diseases

A growing body of evidences links microbiota and bacterial translocation with multiple diseases, including several autoimmune disorders (Mu et al., 2015, 2017a). Due to its strong modulatory effects on host microbiota and immune responses with almost no safety concerns, *L. reuteri* is a good candidate for disease prevention and/or treatment. Indeed, the therapeutic potential of various *L. reuteri* strains has been studied in diverse diseases and the results are promising in many cases.

### Early-Life Disorders

Taking advantage of the safety and tolerance of *L. reuteri* in infants and young children, a lot of efforts have been made to test the potential application of *L. reuteri* against disorders early in life (**Table 3**). In general, the results are promising. *L. reuteri* has been demonstrated beneficial in the prevention and/or treatment of many conditions including diarrhea, functional abdominal pain, caries, atopic dermatitis, allergy, feeding intolerance, and regurgitation. Infant colic, for example, has been the major therapeutic target of *L. reuteri* (**Table 3**). Infant colic is characterized by immoderate crying and affects 10–30% infants (Mi et al., 2015). The exact cause and efficient treatment of this condition have remained elusive. The clinical efficacy of *L. reuteri* DSM 17938 has been demonstrated as most of the clinical trials were successful (**Table 3**). The failure of some studies may be explained by the differences in the dosage of *L. reuteri*, the infant age when the studies initiated, or the baseline microbiota structure. It is worth mentioning that *L. reuteri* is naturally contained in human breast milk (Soto et al., 2014), though inconsistencies exist among individuals. The presence of *L. reuteri* in milk may complicate the results of studies that involved breastfeeding. When given during pregnancy, *L. reuteri* did not show a significant effect on allergy and eczema in infants after they were born (**Table 3**).

**Table 3:** Effects of *L. reuteri* on early-life diseases.

Target	Strain	Duration	Subjects	Result	Citation
Early caries lesions	DSM 17938, ATCC PTA 5289	3 months	Adolescent	No significant change in fluorescence or lesion area	Keller et al., 2014
Caries	ATCC 55730	First year life	Infants	Reduced prevalence of caries and gingivitis score when the kids were 9 years old	Stensson et al., 2014
FAP	DSM 17938	4 weeks	FAP children	Significant decrease in the frequency and intensity of functional abdominal pain	Weizman et al., 2016
FAP	DSM 17938	4 weeks	FAP children	Significant reduction of pain intensity	Romano et al., 2014
Infectious diarrhea	DSM 17938	5 days	Children	Safe and well-tolerated; decreased duration of diarrhea	Dinleyici et al., 2015
Rotavirus diarrhea	-	Up to 5 days	Young children	Shortened diarrhea duration and large decrease in the occurrence of watery diarrhea	Shornikova et al., 1997a,b
Nosocomial diarrhea	DSM 17938	During hospital stay	Children	No effect on the overall incidence of diarrhea, including that related to rotavirus infection	Wanke and Szajewska, 2012
Acute diarrhea	DSM 12246 (with 19070-2)	5 days	Children patients	Decreased duration of diarrhea; decreased period of rotavirus excretion	Rosenfeldt et al., 2002a,b
Acute diarrhea	DSM 17938	3 days	Children	Decrease in diarrhea frequency, duration, and relapse	Francavilla et al., 2012
Acute diarrhea	DSM 17938	5 days	Hospitalized children	Effective decrease of the duration of acute diarrhea	Dinleyici et al., 2014
Diarrhea	ATCC 55730	12 weeks	Infants	Fewer and shorter diarrhea episodes	Weizman et al., 2005
Diarrhea	DSM 17938	6 months	Children	Reduced incidence of diarrhea	Agustina et al., 2012
Diarrhea	DSM 17938	3 months	Children	Decrease in diarrhea episodes and duration; Benefits against respiratory infection	Gutierrez-Castrellon et al., 2014
Infant colic	DSM 17938	3 weeks	Breastfed infants	Significant reduction in crying time	Mi et al., 2015
Infant colic	DSM 17938	3 weeks	Breastfed infants	Reduction in crying and fussing time	Chau et al., 2015
Infant colic	DSM 17938	12 weeks	Newborns	Effective preventive and protective action	Savino et al., 2015a
Infant colic	ATCC 55730	28 days	Breastfed infants	Significantly improvement of colicky symptoms compared with simethicone	Savino et al., 2007
Infant colic	DSM 17938	21 days	Breastfed infants	Improved symptoms; Increase of *Lactobacilli* increase and decrease of *E. coli* in the fecal microbiota	Savino et al., 2010
Infant colic	DSM 17938	21 days	Colicky infants	No effect on the global microbiota composition	Roos et al., 2013
Infant colic	DSM 17938	21 days	Breastfed infants	Higher rate of responders and reduced median crying time	Szajewska et al., 2013
Infant colic	DSM 17938	1 month	Infants	No effect on crying time	Sung et al., 2014
Infant colic	DSM 17938	90 days	Infants	Significant reduction of the mean crying time	Indrio et al., 2014
Infant growth	DSM 17938	98 days	Healthy infants	Well-tolerated but no improvement on growth	Cekola et al., 2015
Atopic dermatitis	DSM 122460 (with 19070-2)	6 weeks	AD children	Improvement of eczema; more pronounced in allergic patients	Rosenfeldt et al., 2003, 2004
Atopic dermatitis	ATCC 55730	8 weeks	AD children	Positive modulation of cytokine pattern in the exhaled breath condensate	Miniello et al., 2010
Eczema	ATCC 55730	-1 to 12 month old	Infants with family Allergic history	No protection of the general occurrence of eczema; Prevention of IgE-associated eczema	Abrahamsson et al., 2007
GI motility	-	30 days	Newborns	Faster gastric emptying	Indrio et al., 2009
Respiratory allergy	ATCC 55730	-1 to 12 month old	Infants	No effect on the prevalence of asthma, eczema or other allergic diseases later in life	Abrahamsson et al., 2013
Feeding intolerance	DSM 17938	Until out of NICU	Preterm infants	Decrease in feeding intolerance and duration of hospitalization	Rojas et al., 2012
Necrotising enterocolitis	DSM 17938	Until discharge	Preterm infants	No effect on NEC rate; Decrease in feeding intolerance and duration of hospital stay	Oncel et al., 2014
Regurgitation	DSM 17938	28 days	Infants	Prevention of regurgitation during the first month of life	Garofoli et al., 2014
Regurgitation	DSM 17938	90 days	Infants	Significant reduction of the mean number of regurgitation	Indrio et al., 2014

*FAP, functional abdominal pain; NICU, neonatal intensive care unit; NEC, necrotising enterocolitis.*

### Systemic Lupus Erythematosus

The SLE is a multi-system autoimmune disease that involves both genetics and environment as the major disease causative factors (Tsokos, 2011; Edwards et al., 2017). The role of gut microbiota in SLE development was suggested by recent studies, and probiotics have been proposed as potential immunoregulators in SLE (Mu et al., 2015, 2017b; de Oliveira et al., 2017; Edwards et al., 2017; Esmaeili et al., 2017). We reported a significantly decreased level of *Lactobacillaceae* in lupus-prone MRL/lpr female mice compared to healthy control mice both before and after the disease initiated in MRL/lpr mice (Zhang et al., 2014). Moreover, we found that treatment with retinoic acid improved kidney disease in MRL/lpr mice, and that the improvement of lupus symptoms was associated with restoration of *Lactobacilli*. This suggests a possible beneficial effect of *Lactobacilli* in lupus. Therefore, we treated MRL/lpr mice with a mixture of five strains of *Lactobacilli* to determine their therapeutic benefit. As anticipated, increasing *Lactobacilli* in the gut improved renal function, reduced serum autoantibodies, and prolonged the survival of MRL/lpr mice (Mu et al., 2017c). Interestingly, *L. reuteri* and an uncultured *Lactobacillus* sp. accounted for > 99% of the observed effects. It suggests a central role of *L. reuteri* in attenuating lupus nephritis. Furthermore, we found that MRL/lpr mice had a “leaky” gut during disease progression, whereas *Lactobacillus* treatment enhanced the intestinal barrier function in these mice and subsequently decreased metabolic endotoxemia (Mu et al., 2017c). At the same time, the local and systemic pro- and anti-inflammatory network was rebalanced by *Lactobacillus* treatment. Specifically, IL-10 production was enhanced while the level of IL-6 was decreased systemically. Strikingly, the benefits of *Lactobacilli* were only observed in females and castrated males but not in intact males. Coincidently, the relative abundance of *Lactobacilli* in gut microbiota did not decrease as disease progressed in male MRL/lpr mice (Zhang et al., 2014). Consistent with our observations, daily consumption of *L. reuteri* BM36301 significantly lowered serum TNF level in females but not in males (Lee et al., 2016). The high serum level of testosterone in males may have led to the difference in the response to *L. reuteri*. Together, these results suggest possible interaction between sex hormones and gut microbiota in autoimmune disease development (Markle et al., 2013; Yurkovetskiy et al., 2013). Further investigation of this link is required. In another lupus mouse model, NZB/W F1, the administration of two *L. reuteri* strains, together with one *L. paracasei* strain, was shown to be effective in ameliorating lupus hepatitis (Hsu et al., 2017). Liver abnormalities, manifested as increased liver enzymes, portal inflammation and histopathological changes, have been observed in both lupus mouse models and SLE patients (Hsu et al., 2008; Grover et al., 2014). In this study, the oral *L. reuteri* treatment largely mitigated hepatic apoptosis and inflammation, suggesting a protective function of *L. reuteri* against lupus-associated liver disease (Hsu et al., 2017). The protection seems to rely on the capability of *L. reuteri* to increase antioxidant activity and reduce cytokines associated with more severe lupus, such as IL-6 and TNF (Tzang et al., 2017). Interestingly, within these two *L. reuteri* strains, only GMNL-263 can significantly promote the differentiation of Treg cells, again emphasizing the uneven immunoregulatory abilities of different *L. reuteri* strains.

### Obesity

The correlation between gut microbiota and obesity is well documented (Okeke et al., 2014; Harakeh et al., 2016). The microbiota composition varies between lean and obese individuals, and a surprisingly high level of *Lactobacillus* spp. has been found in the microbiota of both obese adults and obese children (Armougom et al., 2009; Bervoets et al., 2013). Among different *Lactobacillus* spp., *L. reuteri* was specifically described to be associated with obesity (Million et al., 2012, 2013a). The association was further established when vancomycin-resistant *L. reuteri* in gut microbiota was determined as a body weight gain predictor during vancomycin treatment (Million et al., 2013b). However, in a randomized, double-blind and placebo-controlled clinical trial, the administration of *L. reuteri* JBD301 for 12 weeks significantly reduced body weight in overweight adults (Chung et al., 2016). Moreover, supplementation of infant formula with *L. reuteri* did not increase weight gain in infants (Braegger et al., 2011; Koleva et al., 2015). These conflicting results indicate that *L. reuteri* may influence the development of obesity in a strain-dependent manner. This hypothesis is partially verified in an animal study. In that study, three different strains of *L. reuteri* were used to test their influence on diet-induced obesity (Fåk and Bäckhed, 2012). It was demonstrated that only *L. reuteri* PTA 4659 efficiently reduced the body weight of mice fed with high-fat diet (HFD), whereas *L. reuteri* L6798-treated mice even gained some weight. The changes of adipose and liver weights were consistent with the body weight change.

In animal studies, several strains of *L. reuteri* have been reported to negatively regulate the development of obesity (Dahiya et al., 2017). In addition to the beneficial effect of *L. reuteri* JBD301 to human obese patients mentioned earlier, the favorable role of this strain of *L. reuteri* against weight gain was confirmed in HFD-fed mice (Chung et al., 2016). In HFD-induced obese mouse models, the beneficial role of *L. reuteri* GMNL-263 was also noted (Hsieh et al., 2016). Treatment with *L. reuteri* GMNL-263 reduced the body weight as well as the percentages of adipose tissue and liver to body weight. Interestingly, heat-killed GMNL-263 appeared to have a very similar beneficial function (Hsieh et al., 2016; Liao et al., 2016). *L. reuteri* 6475 has also been shown to be beneficial against obesity in mice (Poutahidis et al., 2013b). The function of *L. reuteri* 6475 was suggested to be largely dependent on its capability to induce Treg cells without changing the gut microbial ecology. Furthermore, the weight loss properties of some reagents have been attributed to their abilities to increase *L. reuteri* in mice. Polymannuronic acid, for example, was able to increase the relative abundance of *L. reuteri* and significantly reduce HFD-induced body weight gain (Liu F. et al., 2017). Whether the increase of *L. reuteri* is the cause of weight loss requires further investigation.

### Neurodevelopmental Disorder

Exposure to maternal obesity *in utero* increases the chance of neurodevelopmental disorders, such as autism spectrum disorder, in children (Connolly et al., 2016). In a recent mouse study, maternal HFD (MHFD) was shown to induce social deficits in the offspring (Buffington et al., 2016). The impaired social ability in GF mice was restored by fecal microbiota transplantation from offspring with maternal regular diet (MRD) but not MHFD, suggesting a potential role of microbiota in this process. Further analysis showed that the abundance of *L. reuteri* was reduced more than ninefold in the gut microbiome of MHFD vs. MRD offspring. The social defects in MHFD offspring were rescued by direct *L. reuteri* administration, suggesting an effect of *L. reuteri* in regulating neurodevelopment in MHFD mice. This regulatory function of *L. reuteri* was attributed to its capability to increase the level of oxytocin (Poutahidis et al., 2013a; Buffington et al., 2016). The results of these studies suggest a potential application of *L. reuteri* in the treatment of patients who suffer from neurodevelopmental disorders.

### Stressor Exposure and Enteric Infection

The composition of gut microbiota shift when the host is exposed to stressors (Bailey et al., 2010; Galley et al., 2014). In C57BL/6 males, social stressors led to an altered intestinal microbiota composition, though there was no significant change in community diversity (Galley et al., 2014). Further analysis showed stressor-induced reductions in the families *Porphyromonadaceae* and *Lactobacillaceae*, especially in the genus *Lactobacillus*. Among *Lactobacillus spp., L. reuteri* was specifically measured and a lower abundance of *L. reuteri* was evident in stressor-exposed CD-1 mice but not C57BL/6 mice. In fact, the level of *L. reuteri* in C57BL/6 male mice was below the detection limit with or without stressor exposure (Galley et al., 2014). It is important to note that stressor exposure increased the severity of *Citrobacter rodentium*-induced inflammation in the gut (Bailey et al., 2010; Mackos et al., 2016). The colonization of *C. rodentium* was promoted by stressor exposure, which subsequently resulted in more severe colonic pathology and increased production of inflammatory cytokines and chemokines (Mackos et al., 2016). Further studies revealed that stressor-induced *C. rodentium* colitis was C-C motif chemokine ligand 2 (CCL2)-dependent. Interestingly, administration of *L. reuteri* ATCC 23272 was able to reverse stressor-induced *C. rodentium* infection, which also relied on CCL2 (Mackos et al., 2013, 2016). However, *L. reuteri* was not able to restore the gut microbiome altered by social stressors. This indicates that the beneficial effect of *L. reuteri* on stressor exposure and subsequent enteric infection is not microbiota-dependent (Galley et al., 2017).

## Conclusion

There has been a decrease in the abundance of *L. reuteri* in humans in the past few decades likely caused by the modern lifestyle (Antibiotic use, western diet, improved hygiene). Such decrease coincides with higher incidences of inflammatory diseases over the same period of time. While evidence is lacking to establish the correlation, it may be helpful to increase *L. reuteri* colonization and/or facilitate its probiotic functions as a new and relatively safe strategy against inflammatory diseases. In addition, through direct regulation or indirect modulation via the host microbiota, *L. reuteri* plays an impressive role in eliminating infections and attenuating both GI diseases and diseases in remote tissues. The safety and tolerance of *L. reuteri* has been proven by the numerous clinical studies. There are multiple *L. reuteri* strains with different host origins, and many of the probiotic functions of *L. reuteri* are strain-dependent. Therefore, it may be advantageous to combine different strains of *L. reuteri* to maximize their beneficial effects.

## Author Contributions

QM and VT wrote the first draft of the review. XL edited and finalized the manuscript.

## Conflict of Interest Statement

The authors declare that the research was conducted in the absence of any commercial or financial relationships that could be construed as a potential conflict of interest.
